# Mapping the Potential of Microfluidics in Early Diagnosis and Personalized Treatment of Head and Neck Cancers

**DOI:** 10.3390/cancers15153894

**Published:** 2023-07-31

**Authors:** Sangeeth Pillai, Jan C. Kwan, Fares Yaziji, Hanwen Yu, Simon D. Tran

**Affiliations:** McGill Craniofacial Tissue Engineering and Stem Cell Laboratory, Faculty of Dental Medicine and Oral Health Sciences, McGill University, Montreal, QC H3A 0C7, Canada; sangeeth.pillai@mail.mcgill.ca (S.P.); jan.kwan@mail.mcgill.ca (J.C.K.); fares.yaziji@mail.mcgill.ca (F.Y.); han.w.yu@mail.mcgill.ca (H.Y.)

**Keywords:** head and neck cancers, microfluidics, personalized medicine, biosensing, tumor-on-chip, high throughput screening

## Abstract

**Simple Summary:**

Head and neck cancers (HNCs) are a group of cancers that affect parts like the mouth, throat, and glands in the neck. These cancers are quite complex and can be challenging to detect and treat early. Microfluidic devices are miniature chips that use a small volume of biological samples and have helped in understanding these cancers better. They have revolutionized the way we diagnose and treat cancer. These devices have also allowed us to create models of individual patients’ cancers on a chip, which help develop personalized treatments. In this review, we will talk about how microfluidics has improved the screening of drugs for treating HNC and how it helps in detecting cancer early and in understanding how treatments work. We will also discuss the challenges we face in using these microfluidic technologies in real-life medical settings.

**Abstract:**

Head and neck cancers (HNCs) account for ~4% of all cancers in North America and encompass cancers affecting the oral cavity, pharynx, larynx, sinuses, nasal cavity, and salivary glands. The anatomical complexity of the head and neck region, characterized by highly perfused and innervated structures, presents challenges in the early diagnosis and treatment of these cancers. The utilization of sub-microliter volumes and the unique phenomenon associated with microscale fluid dynamics have facilitated the development of microfluidic platforms for studying complex biological systems. The advent of on-chip microfluidics has significantly impacted the diagnosis and treatment strategies of HNC. Sensor-based microfluidics and point-of-care devices have improved the detection and monitoring of cancer biomarkers using biological specimens like saliva, urine, blood, and serum. Additionally, tumor-on-a-chip platforms have allowed the creation of patient-specific cancer models on a chip, enabling the development of personalized treatments through high-throughput screening of drugs. In this review, we first focus on how microfluidics enable the development of an enhanced, functional drug screening process for targeted treatment in HNCs. We then discuss current advances in microfluidic platforms for biomarker sensing and early detection, followed by on-chip modeling of HNC to evaluate treatment response. Finally, we address the practical challenges that hinder the clinical translation of these microfluidic advances.

## 1. Introduction

Head and neck cancers (HNCs) represent a heterogenous group of tumors originating from the head and neck regions which comprise the upper aerodigestive tract [1]. Even with an increasing awareness regarding human papilloma virus (HPV)-associated oral cancer and decline in tobacco use-related cancer, HNCs remain the seventh most common form of cancer with more than 650,000 new cases every year, killing nearly 300,000 people annually [2]. The recent increase in the incidence rates of HNCs is chiefly attributed to a rise in oropharyngeal cancer and their associated deaths [3]. These statistics point towards an active rise in new cases and a passive rate of survival in the prevalent population. Among the various types of HNCs, the squamous cell carcinomas originating from the mucosal surfaces of the head and neck regions (e.g., oral cavity, sinonasal cavity, pharynx and larynx) pose the greatest threat [4,5]. This is attributed to the genetically heterogenous and aggressive nature of head and neck squamous cell carcinomas (HNSCCs) [6]. A large part of the molecular characterization of HNSCCs and several other predominant cancer types have evolved in the past couple of decades [7,8]. Recent advances in our understanding of the cancer pathophysiology via multi-omics profiling have led to the identification of new targets which is paving the way for therapeutic advances [9,10,11]. However, these milestones are yet to be translated into clinical success in treating aggressive forms of HNCs [12].

**Figure 1:**
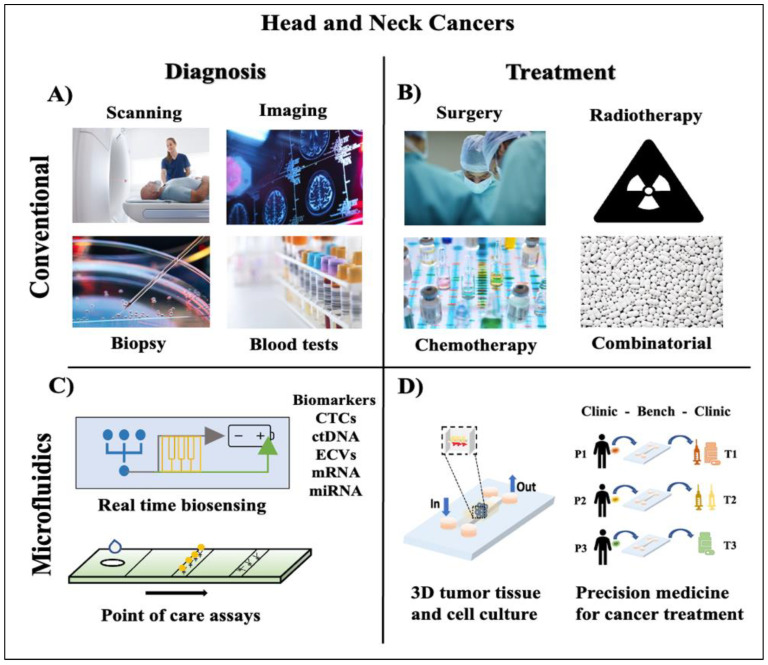
Conventional and microfluidic approaches in HNC diagnosis and treatment. (**A**) Conventional methods of HNC diagnosis include scanning and imaging techniques like computed tomography (CT), magnetic resonance imaging (MRI), nuclear and bone scan, positron emission tomography (PET) scans, ultrasounds, and X-rays. Biopsies include solid tumor and liquid biopsies using needles or endoscopy. Other tests include routine physical/oral examinations, blood tests, and urine analysis. (**B**) Conventional HNC treatment strategies include surgery, radiotherapy, chemotherapy, and combinatorial approaches such as immunotherapies and targeted therapies. (**C**) Microfluidic approaches to HNC diagnosis include microfluidic devices with integrated biosensors to detect, isolate, and enrich cancer biomarkers for early diagnosis. Optimized point-of-care (POC) assays targeted to a specific population can be made available in clinics to perform routine checkups to detect abnormal biomarkers in patient samples for early diagnosis. (**D**) Microfluidic cell culture systems can be used to create personalized 3D tumor-on-chips to test the dynamic effects of drug combinations on patient cancer cells for precise clinical cancer treatment.

Current treatment strategies for HNCs include complex treatment regimens delivered by multidisciplinary teams. Surgical resection and radiotherapy (RT) forms the basis of primary treatment and have shown satisfactory outcomes (Figure 1A,B) [13]. However, most patients with HNSCC are diagnosed once the cancer has reached a locally advanced stage (stage III or IV mainly) making it challenging to treat [14]. For such advanced cases, chemoradiotherapy is the treatment of choice, but in cases of large locally spread tumors, treatment includes organ preservation strategies with chemotherapy (CT), cetuximab-RT (bio-RT) combination, and surgery [15,16]. For advanced and recurrent cases of HNSCC, CT remains the most common line of treatment, but the disease control rate is low (~40%), and in the long term, patients suffer from acute and chronic toxicities drastically depriving their quality of life [14,17]. The advent of immunotherapy has revolutionized the approach towards managing recurrent cases of HNSCCs. Nevertheless, several clinical trials currently exist to evaluate the most efficient combinatorial approach using patient-specific tumor vaccines, such as HPV vaccines, T-cell therapies, and other immunomodulatory drugs to treat aggressive forms of HNCs [1]. Despite this growth in the number of clinical trials at targeted therapies, the recurrence and metastatic nature of HNC, more specifically the HNSCC with a constantly evolving genomic map and drug resistance, poses a significant challenge in improving the overall survivability in the affected population [18]. Given the dynamic nature of the problem, the development of robust, yet versatile preclinical platforms to study individualized cancer biology can serve as a promising approach to combat the heterogenetic and complex nature of HNSCC and assist in devising new therapies [19].

Supported by the advances in material biology, cell culture techniques, and functional assays, researchers have transitioned from the conventional 2D (two-dimensional) systems to study cancer biology. These advances shed light into a plethora of investigations on cancer microenvironment or tumor microenvironment (TME) by utilizing 3D (three-dimensional) tissue cultures including co-culture systems [20,21,22]. Bioengineered tumor spheroids and organotypic models have played a pivotal role in our understanding of TME and serve as versatile tools in modeling multicellular microtissues in tunable matrices. Over the last five years, approximately 74% of research studies in the field of HNCs have predominantly concentrated on utilizing spheroid-, scaffold-, and organoid-based cultures for 3D modeling [23]. Indeed, these complex spheroid models have added a wealth of information to our understanding of TME and the differential drug sensitivity between 2D monolayer and 3D cultures, including xenograft models [24,25]. While cancer cell lines and stem cell-based spheroid and organoid cultures respectively allow the cost-effective analysis of cancer biology, it still remains unprecedented to only rely on these outputs to predict long-term clinical outcomes [26]. In this context, 3D tumor co-cultures offer a superior system in terms of recapitulating tumor–stroma interactions, which is key in activating the TME, in turn promoting tumor growth [27]. For example, Magan and colleagues co-cultured HNSCC patient-derived cancer cell lines with cancer-associated fibroblasts to evaluate the treatment response and changes in phenotypic cell characteristics [28]. They reported an increase in tumor cell proliferation, anti-epidermal growth factor receptor (EGFR) expression, and epithelial to mesenchymal transition when HNSCCs were cultured with cancer fibroblasts. In addition, advances in 3D (bio) printing approaches further provide flexibility in terms of architectural control over the development of multi-matrix, multicellular cancer spheroids [29]. These advances also allow the use of patient-derived HNC cells with a decellularized extracellular matrix (dECM) or bio-printable inks to generate organotypic models to study the mechanical characteristics and treatment response in HNCs [30,31].

Developing sophisticated primary cell-based 3D models for studying the TME is a complex task. However, the challenge lies in utilizing these models for large-scale screening under various parameters, as limited sample availability and a lack of precise control over cell–cell and cell–stromal interactions make it difficult to achieve. Some of these challenges can be overcome by utilizing the rapidly emerging field of microfluidics. Microfluidics is an interdisciplinary field focused on manipulating and controlling small volumes of fluids in microscale channels and chambers comparable to biological cells and microorganisms [32,33]. Its ability to handle small fluid volumes enables precise control, leading to improved experimental control and efficiency [34]. By miniaturizing and integrating fluidic components, such as pumps, valves, and sensors onto a single chip, microfluidic devices offer significant advantages including multiplexing, reduced sample and reagent consumption, faster reaction times, and increased automation [35].

Microfluidics has emerged as a promising technology in cancer research and therapy, offering unique opportunities to improve diagnostics, understand tumor biology, and develop personalized treatment strategies [36,37,38]. The application of microfluidics in cancer spans across various areas including detection, analysis, drug delivery, and monitoring of therapeutic responses [37]. They have made a great impact in the development of diagnostic tools for early screening and diagnosis of HNCs (Figure 1C). Additionally, in studying tumor biology, microfluidic models provide a unique platform to replicate the complex TME using the inventive organ-on-chip approach [39] (Figure 1D). These models can incorporate multiple cell types, simulate physiological conditions, and mimic tumor heterogeneity, allowing for a more accurate representation of cancer behaviour while maintaining a high throughput nature. Microfluidics also plays a crucial role in personalized medicine and allows precise drug delivery, controlled release, and real-time monitoring of therapeutic responses, facilitating more effective and personalized therapies [40]. They enable rapid and cost-effective analysis of genetic mutations, gene expression patterns, and drug responses, facilitating the selection and improved predictability of optimal treatment regimens as opposed to simple 3D models.

In this review, we first describe the scope of microfluidics in precision oncology with a focus on HNCs and discuss their potential in allowing early diagnosis and for high content/throughput screening applications. Next, we discuss the current advances in microfluidic platforms for diagnosis and detection of HNCs with a focus on circulating tumor cells (CTCs) and cancer biomarkers. We then delve into the recent advances in microfluidics in HNC tumor-on-chip platforms for studying therapeutic responses. Finally, we conclude by discussing the challenges in translating these microfluidic proof-of-concept approaches into a clinical setting and provide recommendations for future research.

## 2. Microfluidics, a Peephole into Precision Oncology

Analogous to the notion “one size fits none”, commonly used in the clothing industry, treating cancer using an “all purpose” approach has become obsolete. The ideology of precision medicine surfaced over two decades ago with a principle of understanding the molecular alterations in a patient’s tumor to administer agents that target these changes to improve the clinical management of cancer. Primary studies on these alterations were mostly measured as genomic mutations, which were used as targets to treat and predict patient outcomes [41]. However, while these studies have had a real impact in treating cancer, the associated long-term success, in terms of predictability and clinical management was limited [42]. Additionally, these studies were also challenged by limitations in integrating technology while preserving biology and a lack of robust functional outputs to measure treatment combinations [43,44]. Biomarker-based therapies lie under the umbrella of precision medicine and utilize DNA, RNA, or proteins to identify gene mutations, DNA methylations, protein expressions, etc., to predict disease progression [45]. To date, for the treatment of recurrent and metastatic HNSCC, none of the currently approved agents (cetuximab targeting EGFR [46], pembrolizumab [47], and nivolumab [48] which targets programmed cell death protein-1 (PD-1)) are biomarker-based [49].

Fostered by the advances in high-resolution assays such as single-cell RNA and DNA sequencing, spatial transcriptomics, mass cytometry, metabolomics etc., we can now achieve a significant understanding of the evolving molecular map of heterogenous cancer cells to find potential targets and therapies [50,51]. To add on, the development of robust functional assays that quantify the dynamic effects of different treatments on patient-derived cells serves as an invaluable tool towards improving clinical predictability and thereby effective treatment plans.

Combining functional assays with capabilities of single-cell analysis when performed under physiologically relevant culture conditions opens major avenues in precision oncology. Nevertheless, even with the advances in functional readouts and sequencing technologies, challenges exist in terms of enriched isolation, relevant culture, and maintenance of cancer tissues and cells. The feasibility of using cancer cell lines or primary cells under 2D culture conditions is compelled by their ease, low cost, and high-throughput nature. However, these conditions barely recapitulate the complex TME, especially their multicellular nature and supporting stroma—which is highly sensitive to external physico-chemical and mechanical cues.

Microfluidic platforms possess the ability to bypass many of these challenges and enable the development of highly relevant models, representative of in vivo conditions to facilitate precision therapy. First, microfluidic channels utilize sub-micron liter sample volumes, which makes it easier to run multiple, comprehensive analyses when there is a shortage of relevant sample type (e.g., CTCs, cell-free (cf) DNAs). On-chip platforms such as Parsortrix [52] and CTC-iChip [53] have shown the ability to enrich and isolate viable CTCs, while microfluidic micropillar arrays [54] have been used to directly culture CTCs for use in functional screening. Several groups have shown the use of microfluidic chips for enrichment and characterization of HNC-derived CTCs both from human and mice samples [55,56,57]. Isolated CTCs can be further used with microfluidic microarray chips to run high-throughput, parallelized drug screening to evaluate the patient-specific efficacy of the drug and predict a clinical treatment plan. Apart from CTCs, liquid biopsy samples from HNC patients provide noninvasive access to crucial biomarkers such as circulating tumor (ct) DNAs, microRNAs (miRNAs), proteins, exosomes, and extracellular vesicles [58,59]. In this context, Gwak and colleagues designed an integrated microfluidic device for on-chip extraction and enrichment of cfDNA from blood samples in just 19 min using a 400 µL sample volume which was far superior compared to conventional methods [60].

Another advantage of using microfluidic technology is their versatility as POC devices. A POC test (POCT) is a simple, portable, miniaturized device used for analysis, detection, and/or screening of medical conditions in real time [61]. These devices provide significant advantages over routine screening techniques as they are economical and do not require expensive large equipment or trained personnel to use. The establishment of reliable biomarkers and the recent advances in microfluidic biosensing technologies can be combined to develop specific and sensitive POC devices for routine clinical screening of HNCs. Malhotra et al. created an electrochemical microfluidic array with exceptional sensitivity to accurately detect a four-protein biomarker panel, which was then validated for precise oral cancer diagnosis. The study achieved unparallel detection sensitivity in the range of 5–50 fg·mL^–1^, enabling the simultaneous measurement of interleukin 6 (IL-6), IL-8, vascular endothelial growth factor (VEGF), and VEGF-C proteins in diluted serum [62]. More recently, Soares and team developed a low-cost genosensor-based microfluidic device for the detection of HPV 16 to aid in the early screening of HPV-based HNCs [63]. Similarly, other groups have designed microfluidic devices to capture and sense known proteins, biomarkers, and viruses such as HPVs to diagnose HNCs [62,63,64,65]. These microfluidic POC devices can be optimized and continuously customized as required, and can be translated to clinical setups thereby assisting in early diagnosis, predicting prognosis, and helping in precision clinical management [66,67]. Common biosensing strategies used in these devices can be categorized into three major groups based on their detection principle: electrochemical, optical, and mechanical sensing [68]. Enzyme-Linked Immunosorbent Assay (ELISA), Polymerization Chain Reaction (PCR), Surface-Enhanced Raman scattering (SERS), fluorescence-enabled and microbead-based assays are some of the major techniques routinely used for diagnostic and screening application of molecular analytes [69,70]. Detailed descriptions of these biosensing principles and their applications as POC devices can be found here [68,69]. Nevertheless, despite the tremendous potential, currently, there are no commercially available, POC microfluidic devices for early screening of HNCs, probably due to the lack of validation of biomarker specificity and sensitivity and associated regulator issues. The most common strategies commercially available for clinical oral cancer screening involve the use of fluorescence-based light to visualize cancerous lesions. A few commercially available microfluidic technologies optimized for other cancer types are listed in Table 1. However, these kits are still not fully optimized for use as POCT for routine clinical diagnosis. In the near future, these technologies can probably be adapted to be used for other cancer types including HNCs.

The second advantage of using a microfluidic approach towards precision therapy is the possibility of compartmentalization for cancer cell culture. Multichannel microfluidic chips act as effective co-culture systems and allow independent culture of different cell types promoting cell–cell crosstalk. Such culture systems can be established with precise spatio-temporal control and continual circulation aiding in both mechanotransduction (e.g., simulating TME) and delivery of signaling cues (e.g., administration of a chemotherapy agent). Additionally, depending on the fabrication method, microfluidic devices allow precise control over molecular diffusion of chemotactic agents and study cellular migration including vascular biology [40]. This unique ability of microfluidic platforms to closely simulate in vivo-like conditions potentiates enhanced predictability in terms of patient-specific treatment responses. Owing to these benefits, several groups have reported the fabrication of organ-specific cancer or tumor-on-chip platforms to study complex tumor biology, lymphatic and vascular involvement, and drug interactions [74,75,76]. One such model towards personalized medicine in treatment of HNCs was reported by Jin et al., where they created a perivascular tumor-on-chip model to assess the effect of drugs on both cancer cells and the endothelium [77]. They found that while the anticancer drugs at single high doses killed HNC cells, they also caused significant damage to the endothelial cells. Further, they used patient-derived samples to test drug combinations to determine maximum sensitivity and specificity for each patient for use in decision making prior to clinical treatment [77]. Although RT stands among the first line for HNC therapies, patient/tumor response to radiation can be quite variable depending on individual factors unique to each patient. While these models serve as invaluable tools in understanding patient-specific treatment requirements, the low throughput nature (testing few drugs/patient samples) limits the complete utilization of microfluidic potential.

**Figure 2:**
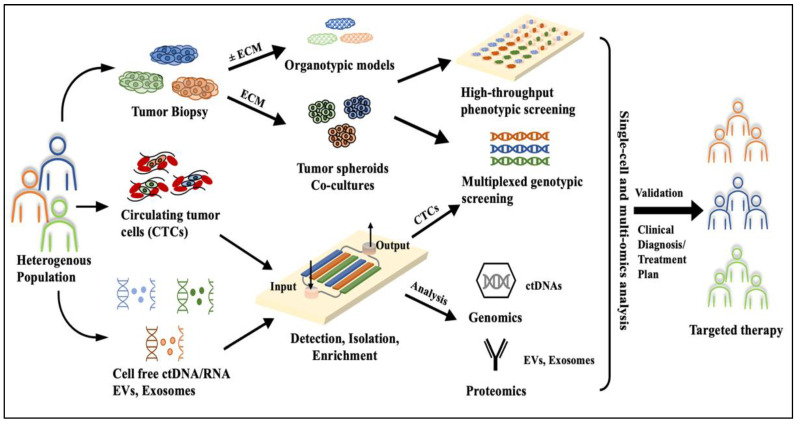
Illustration of a proposed microfluidic workflow towards precision/targeted cancer therapy. Samples from a heterogenous group of patients are collected as solid tumor biopsies or blood/urine samples (liquid biopsies). Tumor tissue biopsies are matrix-embedded and cultured in 3D conditions either outside the chip or on chip with vasculature (endothelial cells) and immune components. Cultured tumor spheroids or organotypic models are transferred to multi-wells (96- or 384-wells) and phenotypically screened with hundreds of small molecules or drug combinations; 3D models can be further genetically screened using loss or gain of function by tools like CRISPR/Cas9 and are subjected to deep sequencing and single-cell multi-omic analysis. Similarly, liquid biopsy samples can be used to perform on-chip detection, isolation, and enrichment of CTCs, which can be subjected to downstream cellular analysis. Simultaneously, cell-free DNA, RNA, proteins, EVs, and exosomes can be monitored in real time with sensor-integrated microfluidic devices.

Functional assays, when used in combination with microfluidic models, provide not only high physiological relevance, but hold the multiplexing potential to test hundreds of drugs or small molecules in conjunction with several patient samples at desired complexity. A schematic proposal of utilizing a microfluidic workflow for precision medicine is shown in Figure 2. First, samples in the form of solid tumor or liquid biopsy, including blood and urine, are collected from patients. Tumor tissues are subjected to enzymatic digestion to obtain single cells or used as organotypic cultures to preserve tissue integrity. In this context, droplet-based microfluidics can be used to obtain single tumor cells, with high efficiency for drug screening and multiplexed analysis [61]. Alternatively, digested cells can be combined with matrices such as Matrigel or other hydrogels in combination with vascular components such as endothelial cells to develop tumor spheroid and organoid co-cultures. Similarly, hundreds of organotypic slices can be obtained from small tissue biopsies using vibratomes and can be either embedded into ECM gels or used alone. Cultured 2D cells or 3D structures (single cells, tumoroids, organotypic models) are seeded into microarrayed microfluidic devices, in the presence of vascular or immune components, and subjected to multi-drug/small molecule screens [78]. Large-scale high- throughput phenotypic screens can be then run on these models under dynamic conditions using high-content screening approaches [79,80]. Simultaneously, multiplexed genotypic screening of 2D or 3D cultured cancer cells can be achieved using technologies like Clustered Regularly Interspaced Short Palindromic Repeats (CRISPR/Cas9) and next-generation sequencing approaches to identify genomic scale target identification [81,82,83].

In parallel, liquid biopsy samples can be used to isolate CTCs, cell-free ctDNA, miRNA, extracellular vesicles (EVs), and exosomes using previously described on-chip platforms to detect, isolate, and enrich CTCs. These CTCs, even when obtained in small quantities, can be incorporated into the 2D/3D culture workflows and subjected to targeted panel sequencing approaches [84]. Cell-free biomarkers can be simultaneously detected by passing them through sensor-integrated microfluidic devices and used downstream for genomic and proteomic analysis [85,86,87]. Further, the most confident drug treatment interactions can be subjected to single-cell multi-omic analysis to analyze in- depth gene regulatory mechanisms and potentially identify novel targets for targeted therapy of HNCs [88,89]. By combining multi-spectral technologies, it is now possible to utilize functional microfluidic assays to assess and quantify, at single-cell resolution, the complex nature of cancer pathophysiology and thereby develop superior personalized treatment regimens.

## 3. Microfluidic Systems for HNC Diagnosis and Screening

How cancer is treated has become a significant driving force of the current technological innovation ranging from nanotechnology, radiosurgery, immunotherapy, and many more [90]. However, it is very important to outline that these treatments’ clinical efficacy and success depend heavily on whether the cancer is in its early to middle or late stages. This is especially true with HNCs due to the aforementioned heterogeneous and aggressive nature of HNSCCs and their origins in a highly vascular location that also neighbors lymph nodes [91]. As a result, early detection and screening of HNCs is a key factor that can drastically improve treatment outcomes and patient survivability [91,92]. Current screening strategies to detect early forms of HNC have been associated with using imaging techniques such as head magnetic resonance imaging (MRI) or sinus contrast-enhanced computed tomography (CT), but issues may arise such as patients being claustrophobic when undergoing MRI for an extended period or that there is a poor assessment of soft tissues and exposure to doses of radiation in CT [93]. Another routine screening method is a biopsy via fine needle aspiration (FNA). However, some limitations of using FNA are that it may produce non-diagnostic or indeterminate cytology results, possess limited tissue access, as well as poor repeatability of tumor sampling which are also all dependent on the physician’s expertise and interpretation [94]. Although FNA is a valuable technique that is safe and minimally invasive, many researchers have begun investigating alternative screening methods including liquid biopsies that can be conveniently collected from a patient’s bodily fluids, such as blood or saliva, to screen and diagnose HNC at a much earlier stage by analyzing key cancer-specific biomarkers [95].

Biomarkers play a crucial role in the early diagnosis of HNCs, as they are specific molecules or indicators that can be detected in various body fluids or tissues, indicating the presence of cancerous or precancerous lesions. Early detection of HNCs is vital for improving treatment outcomes and patient survival rates. Combining microfluidic assays with known biomarkers of HNCs allows for routine screening and early diagnosis in high-risk populations. The rise in HPV-related oral cancer indicates that viral infection is a significant risk factor for certain HNCs, particularly oropharyngeal cancers. Detecting HPV DNA in tumor samples or oral swabs can aid in identifying high-risk patients [96]. The tumor suppressor protein p16INK4a is seen to be overexpressed in HPV-positive HNC patients and can be used as a crucial biomarker in early diagnosis of high-risk HPV-positive patients [97]. Cyclin D1 overexpression is considered a crucial prognostic marker for HNCs, especially with laryngeal and nasopharyngeal cancers [98]. As previously discussed, the most successful targeted therapies for HNSCC is based on EGFR inhibition, especially in advanced and metastatic cases.

Salivary biomarkers such as matrix metalloproteinases (MMPs), cytokines, and microRNAs, have shown promise for early detection of HNCs, as saliva can be collected non-invasively [99]. A systematic review and meta-analysis by Guerra et al. filtered out five key single biomarkers including interleukin 8 (IL-8), choline, pipecolinic acid, l-phenylalanine, and S-carboxymethyl-l-cysteine which show potential for high diagnosis accuracy [100]. Detecting specific mutations or genetic alterations in ctDNAs can aid in early cancer detection and monitoring treatment response. CtDNAs obtained from saliva samples of HNSCCC patients have also shown a high co-relation between hypermethylated gene targets (*RASSF1A*, *DAPK1*, *p16*) [101]. Small non-coding RNA molecules such as miRNA have been proven as some of the most clinically relevant biomarkers for cancers. In HNCs, most investigated miRNAs include miR-21, -375, -99, -34a, -200, -31, -125a/b, -196a/b, -9, -181a, -155, -146a, -23a, -16, -29, and let-7. Detailed information on individual miRNA types seen in different types of HNC can be found in this comprehensive review [102]. While biomarkers hold great potential for early detection of HNCs, extensive, long-term research and validation studies are necessary to establish the efficacy and reliability of their routine clinical use, especially when used with POC devices for screening and detection.

A liquid biopsy approach allows for a non-invasive and accessible screening method for HNC and it can also provide sample repeatability and real-time monitoring at an inexpensive cost [95,103]. In screening for HNC, the most common analytes used from liquid biopsies are CTCs, ctDNAs, EVs, mRNA, and miRNAs [23,104]. Existing technologies used to quantitively evaluate these analytes from liquid biopsies range from flow cytometry, mass-spectrometry, real-time PCR (qRT-PCR), or size-exclusion chromatography (SEC) depending on the analyte [19]. However, the amount of equipment and protocols required to conduct each analysis is time-consuming along with the required lab training to use the equipment resulting in an overall higher cost. Additionally, the ability to regularly monitor multiple key biomarkers in parallel or screen in real-time HNC in numerous patients remains a challenge. Another major limitation of existing screening and detection techniques includes the specificity and sensitivity to detect and isolate low levels of analyte concentrations [19,23,105]. Table 2 illustrates the studies that have employed microfluidic methods utilizing liquid biopsy samples for early detection and screening of head HNCs.

Microfluidic technologies have become a key proponent in mimicking the complex TME as an in vitro 3D model addressing many challenges faced by existing technologies in the screening and detection of HNC [23,131]. Microfluidic systems are small in nature and can be easily handled but are highly sophisticated. These systems can be compartmentalized with interconnected chambers that simultaneously experience in parallel similar physiological factors in the TME such as oxygen concentration, nutrient availability, flow patterns, fluid volume, surface tension, or hydrostatic pressure [132]. These parameters can also be controlled and are tunable, allowing for real-time monitoring of very low levels of analytes from patient-derived cells, prospectively to screen for potential HNC or evaluate how they may interact with certain cancer therapeutic combinations. Additionally, the combination of immunofluorescence staining and the small transparent features of microfluidic systems allows for high-quality imaging under confocal microscopy to further add the ability to screen key biomarkers of HNC in real-time.

Many studies have elucidated many key findings and the efficacy of microfluidic systems in screening and detecting key biomarkers for HNC. Microfluidic systems still have yet to be used in a clinical setting but there has been tremendous groundwork made in developing novel screening and detection methods [133]. One such study by Sharafeldin and colleagues investigated the use of a 3D-printed microfluidic microarray capable of detecting desmoglein 3 (DSG3), a known metastatic biomarker for HNSCC, at ultralow concentrations (0.1 fg/mL) [65]. The authors concluded that using on-chip sonic-assisted chemical lysis and high signal amplification from streptavidin-Poly-HRP labels and ultrabright femto-luminol, achieved sub-femtogram limits of detection of cell-residing proteins in a single cell. Additionally, the use of a 3D chitosan hydrogen film in the detection chambers of the microfluidic chip immobilized and anchored a large number of capture antibodies helping to increase interaction kinetics and sensitivity, as well as decrease assay time. A common difficulty among many microfluidic technology studies to screen for cancer in general is the ability to capture and isolate CTCs due to their small size and potential of adhering to blood cells, also known as circulating hybrid cells. Kulasinghe and colleagues investigated spiral microfluidic chips based on the dean vortex flow to enumerate and characterize CTCs from HNC patients [55]. Utilizing curvilinear microchannels under a continuous flow rate of 1.7 mL/min helped focus CTCs near the microchannel’s inner walls and drove smaller hematologic cells toward the microchannel’s outer walls. This method of size or deformability separation was able to identify single and cluster CTCs. Kwak and colleagues developed a similar spiral-shaped microfluidic channel focusing on capturing magnetically labeled CTCs based on the epithelial cell adhesion molecule (EpCAM) expression level while also separating CTCs from blood cells [113]. Depending on whether CTCs were EpCAM positive or negative, they were sorted into designated cell-trapping segments within the microfluidic chip. Another method of separation includes dielectrophoresis (DEP) which employs the electrophysical properties of CTCs to isolate them under a nonuniform electric field. Montoya Mira and colleagues investigated a label-free technique using a microfluidic dielectrophoretic device to sort and enrich CTCs while separating healthy peripheral blood mononuclear cells (PBMC) [114]. The microfluidic device was composed of a V-shaped electrode able to deflect PBMCs using DEP forces. Cells that experienced stronger DEP forces were sorted to the side outlets while less responsive cells are enriched and focused through the center channel. These separation techniques incorporated into microfluidic devices have been extensively studied with many more examples, however, all of these techniques have been demonstrated to be applicable using different analytes from liquid biopsies to screen for all cancers, especially HNC [134]. However, further research is needed to reliably and repeatedly capture and isolate pure CTCs due to their inherent rarity and heterogeneity.

Another important aspect of microfluidic technology is the ability to reduce the overall cost while maintaining accuracy in the screening of HNC in its early stages. Zoupanou and colleagues took an alternative approach by investigating a low-cost microfluidic device, SMILE, for the early screening of oral squamous cell carcinoma [135]. Their SMILE device consisted of a micromixer developed on two-level microchannels for sample preparation and a serpentine pathway for bio-detection both made out of polymethyl methacrylate (PMMA). Their results demonstrated that a slow flow rate of 2 μL/min and a serpentine microchannel lengthened the path the cells are forced to run on and maximized the possibility for cells to be captured by immobilized antibodies (Anti-EpCAM). Current efforts have been concentrated on ensuring accurate replication of the TME in microfluidic devices and the ability to isolate and capture CTCs, which have all been elucidated to be successful in many studies. One drawback of this success is the reliance on polydimethylsiloxane (PDMS) frequently used to fabricate microfluidic devices. Some limitations include the leaching of oligomers that could contaminate biological samples [136], swelling due to absorption of small molecules [137], the permeability to organic solvents [138], or reproducibility of PDMS microfluidic devices via 3D-printing [139]. The implications of these limitations lay a foundation for the optimization of PDMS or suitable alternatives to PDMS such as hydrogels, thermoplastic polymers, glass, or polyimide [140]. Future research on using potential alternatives to PDMS will need to ensure the repeatability and accuracy of screening and detecting key biomarkers of all cancers in their early stages while maintaining low-cost and efficient fabrication of microfluidic devices to screen for HNC.

Overcoming the challenge of increasing the sensitivity and specificity of microfluidic technology with regard to the screening and detection of key biomarkers of HNC requires a large testing sample population to reliably translate these techniques into a clinical setting [141]. High-throughput screening (HTS) is a technique that works by testing and analyzing large numbers of chemical or biological compounds against specific targets in a very short time, with the goal to develop either drug or diagnostic tools [142,143]. Researchers have observed that many limitations of existing HTP screening systems can be resolved by using microfluidic technologies since they can be produced at a much lower cost and work on liquid volumes at the level of nano- to microliters or smaller [143]. Wang and colleagues employed an HTP screening method using a solid-phase graphene-oxide quantum dots integrated microfluidic immunofluorescence micro assay chip termed GIMIFA [144]. The authors reported that GIMIFA was composed of a water-soluble graphene-oxide quantum dots (GOQD) layer and a capture antibody layer immobilized on the GOQD layer, containing 20 parallel microchannels 20 μm in width. Their observations determined that taking advantage of GOQDs properties of tunable fluorescence, unique π-π orbital interaction on the interface, and fluorescence quenching allowed the capability of achieving strong and uniform immobilization of antibody/antigen barcode arrays and the simultaneous detection of multiple biomarkers at a detection limit of 1 pg/mL. This study has wide practical applications in the early screening and diagnosis of all cancers, yet there is an added benefit of combining the use of HTP screening and microfluidic technology in the modern era, which is the use of artificial intelligence (AI), particularly the subset of machine learning. From the same laboratory, Wang and colleagues had similarly developed a single-cell culture and detection microfluidic chip platform with self-assembled GOQDs paired with a machine-learning strategy, the K-mean strategy [145]. The captured antibody array was microprinted on a GOQD-assembled chip with a PDMS chip to form a multi-index antibody barcode. Utilizing this method allowed for quantitative measurement of secreted biomarkers using fluorescence signals and the authors reported the ability to analyze and classify single-tumor cell secretion data with a recognition accuracy of 95 percent.

As the technology of microfluidic devices improves drastically in the early screening of key biomarkers for HNC, it will be important to combine the use of AI as it can aid in the real-time analysis, provide immediate feedback, and also improve accuracy with the help of specifically designed algorithms that can be trained on large sets of patient data to allow microfluidic devices in detecting specific biomarkers for specific cancers. Additionally, existing isolating and capturing of key biomarkers along with low-cost and consistent fabrication methods of microfluidic devices will be a required step in the practice of personalized medicine.

## 4. Microfluidic Tumor-on-a-Chip Platforms for HNC Therapy

As previously discussed, the treatment of HNCs typically involves the implementation of conventional methods such as surgery, chemotherapy, and RT. These modalities have demonstrated their efficacy, particularly when administered during the initial stages of the disease before significant progression occurs. However, it is important to note that individual patients exhibit varying responses to these treatments, posing a challenge in developing a universally applicable approach. Given the unique nature of HNC, which necessitates a heightened level of accuracy and precision, the integration of microfluidic chip platforms has emerged as a promising avenue for enhancing the effectiveness of cancer treatment interventions. The utilization of these platforms allows for the prediction of treatment responses, ultimately contributing to more efficient applications in the management of cancer.

**Figure 3:**
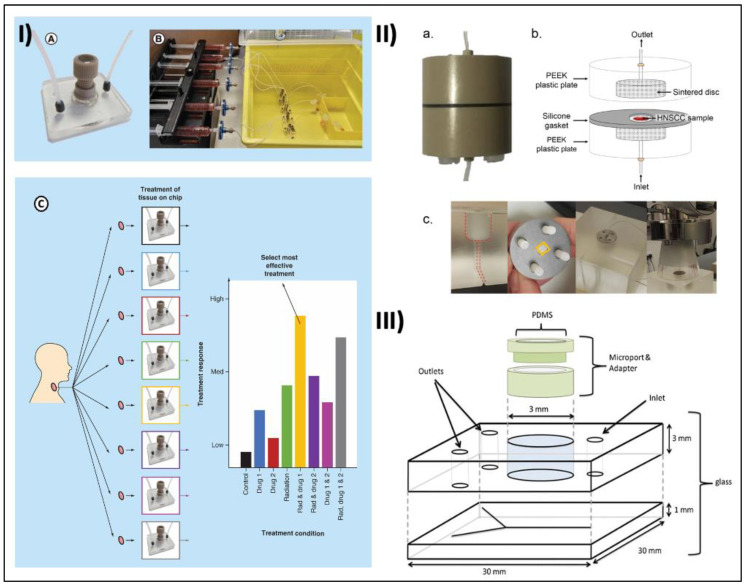
Examples of microfluidic tumor-on-a-chip devices for HNC modeling. (**I**) (**A**) Microfluidic device set up with polyether ether ketone (PEEK) plastic microport and PDMS-filled PEEK-threaded adaptor. (**B**) Complete culture setup with syringes containing complete medium attached to a pressure-driven syringe pump for continuous flow to multiple tissue sample within the device. (**C**) Parallel ex vivo testing of HNSCC tissue on a chip. Reprinted with permission from [146]. (**II**) (**a**) Close- up picture and (**b**) schematic diagram of a PEEK-based tumor-on-a-chip device. (**c**) Irradiation set- up with custom-made Perspex (PMMA) phantom for a reproducible setup and a full-scatter condition to simulate clinical radiation delivery. Reprinted with permission from [149]. (**III**) Schematic figure of glass microfluidic device with access holes and a central chamber (diameter 3 mm) in the top plate. The channel network is etched into the bottom 1 mm glass layer to produce channels of 190 μm width, and 70 μm depth, diverging into two channels. Reprinted with permission from [150]. Copyright (2023), Springer Nature.

In a move toward personalized medicine, maintenance of tumor biopsies in microfluidic tissue culture devices are being developed. Bower et al. reported a microfluidic culture system that successfully maintained HNSCC tissues for 48 h, and the culture system allows testing of different treatment modalities with response monitoring (Figure 3I(A–C)). The chip is designed with microstructures to help manipulate the fluidic environment. The microstructures aid in the analysis of the cultured tumor cells and provide crucial data about the response to the various types of therapy [146]. Immunotherapy is a treatment approach that utilizes the patient’s immune system to kill cancerous cells and is often combined with forms of treatment like chemotherapy and RT. Immunotherapy may involve the use of monoclonal antibodies or the use of checkpoint inhibitors. Testing immunotherapy in in vitro settings has its own challenges, especially as a co-culture of two different cells, i.e., cancer cells and immune cells with different adherent properties. Many microfluidic chip assays were developed to facilitate the in vitro testing of immunotherapy; one of which is a method where cancer cells were stained and suspended in Myogel/fibrin and put in one chamber. Immune cells were stained and suspended in a media supplied with 10% serum and placed in other chambers. Immune cells were then divided into three groups: (1) control (with no drug), (2) PDL-1 antibody, and (3) Indoleamine 2,3-dioxygenase 1 (IDO-1) inhibitor. This setup is closer to an in vivo situation where the patients receive immunomodulatory drugs through intravenous injection. This assay could be used to predict the efficacy of immunotherapeutic drugs for individual patients and show how immune cells migrate to the cancerous cells’ chamber [147].

In addition, to enhance the efficiency of personalized cancer therapy precision testing, a study introduced the multiplexed microfluidic immunohistochemistry (IHC) system that enabled high-throughput analysis of tissue microarray and the response to different treatment modalities. Using the patterns of biomarker barcodes, which consist of a series of expressed linear patterns of specific biomarkers. A multichannel PDMS microfluidic device was reversibly assembled on each core of cell microarray (CMA) section slides. By injecting primary antibodies from different biomarkers independently into each channel, multiplexed immunostaining can be performed on each core of the CMA. The study confirmed that this system, which has been verified by performing multiplexed microfluidic IHC on CMA samples, provides high reproducibility and the potential for high-throughput analysis with molecular diagnostic capability [148].

RT involves using focused radiation to cause nuclear damage to the tumor cells. The extent of nuclear damage to the cells helps to trigger apoptosis leading to the death of the tumor cells. Irradiation damages the DNA within the tumor cells, preventing further replication and triggering cell death. The biocompatible and physical properties of microfluidic chips allow the analysis of tumor cells and their response to different doses of radiation. Carr et al. used a double-layered glass microfluidic device with one inlet and two outlets to maintain HNSCC samples from 35 patients and tested different radiation doses between 2 Gy and 40 Gy to evaluate cell death using levels of lactate dehydrogenase (LDH) and cleaved cytokeratin 18 (cCK18) [151]. They showed that these simple microchips can be practically irradiated like human subjects and be used to assess radiation-induced cell death in HNSCC and therefore has the potential to be used to predict radiation response [151]. Subsequently, in a study by Cheah et al., they used a similar microfluidic platform to maintain HNSCC tumor samples from five early-stage patients and irradiated the tissue inside the device with different (0, 5, 10, 15, and 20 Gy) single doses [152]. In addition to LDH and cCK18, they also evaluated the phosphorylated -H2AX (γH2AX), a marker of double-stranded DNA breaks and Ki-67, a proliferative marker in the tumor tissues. While their study reported a dose-dependent decrease in the percentage of Ki-67-positive nuclei with increasing radiation doses, there was no significant difference in LDH levels between irradiated and control groups [152]. The findings in this study demonstrate discrepancies compared to the previous research conducted by Carr et al., highlighting the significant variations in how patients and tumors respond to different doses of irradiation. These preclinical outcomes obtained using a microfluidic chip offer essential insights into the individualized sensitivity of patients and tumors to radiation. Such information is valuable in the clinical setting for designing appropriate treatment regimens.

More recently, to improve consistency in radiation dose delivery, Kennedy et al. described a microchip assay to optimize the amount of radiation exposure during RT. In their study, each tumor-on-a-chip platform is composed of two identical parts with central axial threaded holes to secure the radiation tubing to the system via coned adaptors (Figure 3II(a–b)). Irradiation was performed using a 120 kVp photon beam simulating clinical cases where cancerous tissue is irradiated at depth (Figure 3II(c)). After using different radiation doses on different microchip platforms, frozen tissue sections were subjected to IHC. The results were reported that fractionated irradiation reduced proliferation and increased DNA damage and caspase-dependent apoptosis (cCK18) compared to control [149].

In cases of recurrent and metastatic late-stage HNCs, chemotherapy plays a critical role in the long-term prognosis and quality of life in patients. In this context, microchip arrays provide personalized chemotherapy in cancer patients. Astolfi and colleagues reported using a microfluidic platform for ex vivo chemosensitivity testing of tumor tissues from a cancer patient using the micro-dissected tissues (MDT) chip method [153]. The MDT chip method was developed with a goal to have a large enough tissue to maintain tissue complexity with high viability and control nutrients, waste, and signaling gradients naturally as well as minimize anoxia at the tissue center. While this study used non-HNC cells and tissues to optimize the microfluidic device and approach, the technology has the potential to be translated in other tumor tissues to improve treatment success rates by identifying potential responders earlier during the course of treatment [153]. Hattersley et al. used a similar approach to test HNSCC tumor biopsies using a glass microfluidic system which enables continuous media perfusion and waste removal, recreating in vivo laminar flow and diffusion-driven conditions (Figure 3III) [150]. Primary HNSCC or metastatic lymph samples were subsequently treated with 5-fluorouracil and cisplatin, alone and in combination, and were monitored for viability and apoptotic biomarker release over 7 days. An apoptotic biomarker, cytochrome c, was analyzed and all treated samples showed higher levels than the control, with the combination therapy showing the greatest effect. This device offered a novel method of studying the tumor environment and offers a pre-clinical model for creating personalized HNSCC treatment regimens [150].

In conclusion, tumor-on-chip microfluidic platforms and functional assays are a valuable tool in the preclinical, in vitro, and ex vivo modeling of HNCs. They offer improved sensitivity, precision, and the ability to analyze small sample volumes and enable testing of multiple drug combinations to evaluate dynamic changes in patient samples in response to treatments. These assays have the potential to improve patient outcomes by enabling early detection, accurate monitoring, and predict personalized treatment strategies.

## 5. Pros and Cons towards Clinical Translation

Traditionally, cancer is often diagnosed through biopsy and the histological evaluation that follows, which could be invasive, risky, and expensive. On the other hand, the use of chips with microfluidic properties allows use of “liquid biopsies”, where blood samples are analyzed for proteins, exosomes, vesicles, and CTCs present in the bloodstream that have broken off from the main tumor/metastasis tumor [38]. These proteins/exosomes, as well as CTCs, could be analyzed for the presence of cancer, and as such, is less invasive and expensive compared to a biopsy, since the operating requirements of these chips could be performed at a very low cost [38]. This is logical because drawing blood is a lot cheaper and could be conducted much more routinely than a biopsy.

Moreover, these chips could automate some processes found in traditional biopsy diagnostic techniques and hence have a smaller requirement in terms of labor intensity and trained technicians as opposed to the biopsy and the IHC that often follows [154]. These on-chip devices could lead to a more personalized treatment for each patient, since the body’s immune responses to eradicate cancer cells, in addition to how those very immune responses are inhibited, varies between different individuals and can also depend on the characteristics of the tumor [155]. Hence, the method of evaluating the cancer’s biomarkers allows for a more tailored planning of therapy by establishing a “tumor profile” [156]. This tumor profile can help clinicians in drug therapy by targeting the tumor more specifically. Although these tumor profiles change with time [156], the analysis using on-chip devices can be performed when needed again due to the lesser cost and minimal invasiveness to evaluate various treatments’ effectiveness. Recent advancements in drug screening have focused on utilizing microfluidic chips to directly analyze patient-derived cells and proteins while preserving their in vivo characteristics. These efforts aim to improve the isolation, purity, yield, and amplification of the target proteins and cells within a shorter timeframe compared to conventional methods, which can be labor-intensive. These developments hold promise for more efficient and streamlined drug screening processes [157]. This further contributes to making personalized treatment more readily available by reducing the time needed to obtain results and the costs associated with the process.

Translating these aforementioned methods to clinic, however, has its own challenges. CTCs, exosomes, extracellular vesicles, as well as other proteins that are present in the patient’s bloodstream and are associated with the tumor in question, are hard to isolate from human peripheral blood since there are a lot of other entities that have a similar size as the target proteins and cells [158]. Thus, further specialized microengineering capabilities are needed, where microfluidic devices can isolate desired proteins and cells efficiently, and have superior detection sensitivity, using integrated biosensors that help with the process. Although there has been good progress, it still remains a challenge to be surmounted completely [159]. The development of these technologies could potentially provide unprecedented opportunities to create a cell culture beyond the current in vitro models by further enhancement in emulating critical tissue-to-tissue interaction. Operating costs of the chips themselves are relatively inexpensive and do not require extensive professional supervision during usage, while the fabrication of these lab-on-a-chip processes involves very precise microengineering techniques on a micro or nano level. Consequently, the equipment required for chip manufacturing can be costly, and the process itself often demands skilled personnel. This can pose challenges for medical institutions in accessing the necessary equipment and poses expertise for chip production [160].

The lack of standardized chip design is a prevalent issue in the field of microfluidics, particularly in the context of lab-on-a-chip systems for cancer diagnosis and treatment. Many studies have developed customized devices based on their specific experimental needs, which limits compatibility and interchangeability between different applications and experimental setups. This lack of standardization hampers the validation and transferability of results across different study settings. To improve efficiency and streamline processes, it is important to establish standardized practices for microfluidic chip fabrication, design, and usage. Without such standardization, operating different chips under varying experimental conditions becomes challenging, requiring repeated optimization and extensive personnel training. Additionally, the lack of communication among academic groups specializing in chip development further limits the scalability of innovative devices emerging from different institutes. Furthermore, the relative novelty of microfluidic lab and tumor-on-a-chip devices means that there are limited individuals trained or equipped to develop and apply these devices.

Efforts should focus on developing a versatile pipeline for microfluidic device design that minimizes training requirements and overcomes physical constraints. Several microfluidic startups and biotechnology firms offer prefabricated, custom-made on-chip devices for different organ systems. Such systems can be designed to iteratively accustom to different cell types and once optimized, are assay-specific. Academic research groups can collaborate with companies to scale up the fabrication of already optimized microfluidic devices and create marketing pipelines to make the technology readily available to more researchers. Collaboration between clinical cancer centers that have routine access to cancer biopsies and research laboratories is crucial and can lead to the identification of novel targets and biomarkers. This association can be extended to industrial research and development teams who can design clinical-grade, biomarker-specific, microfluidic assays and POC devices.

Another challenge with the use of microfluidic devices in analyzing patient samples is the accompanying ethical and regulatory concerns [161]. Firstly, there is a need for informed consent from patients, ensuring their awareness regarding the purpose, associated risks, and potential benefits of using their samples in developing microfluidic devices. Secondly, patient privacy and confidentiality must also be strictly maintained, with appropriate measures taken to protect sensitive data generated during the analysis and data-sharing processes. Additionally, strict regulatory frameworks should be in place to oversee the development, manufacturing, and use of microfluidic devices to ensure their safety, accuracy, and reliability [162]. Further, quality control measures and adherence to ethical guidelines for the handling and disposal of patient samples are imperative to successfully translate the microfluidic findings into the clinics.

## 6. Conclusions and Future Directions

The complex and aggressive portrait of HNCs has led to high morbidity and poor survivability among patients. Microfluidic technologies have shown the potential in replicating the in vivo TME for HNCs, especially aggressive forms like HNSCC, enabling early diagnosis and therapeutic evaluation. Microfluidic technology accurately represents the complexity of tumors by controlling physical and chemical cues in microarchitecture. It integrates well with various sensing and detection methods, making it suitable for early cancer detection, high-throughput screening, and studying the mechanisms of anticancer treatments. Utilizing microfluidic technology to preserve patient biopsies presents a potential alternative to animal models that is not only cost-effective but also easily scalable and user-friendly. These advancements hold the promise of improving scientific research by employing human tissue to better represent human diseases, thereby facilitating more accurate and relevant findings in the future. However, despite the potential, microfluidic technologies are still in the early stages, and further validation through comparison with in vivo results is necessary before clinical implementation. For example, targets and hits identified from the testing of hundreds of drugs using high-throughput microfluidic platforms should be replicated using multiple patient-derived cell lines or cell models. These drug interactions should be further validated for in vitro generalizability by multi-user groups. The most confident hits and targets should then be studied further using in vivo models to confirm their clinical relevance. Such a sequential approach to validation using microfluidic approaches would reduce time-consuming animal studies to screen a large number of drug candidates, and allows a quicker translation to clinics.

Currently, most cancer-on-chip research primarily focuses on demonstrating the capability of microfluidic devices to measure specific analytes (CTCs, proteins, DNA/RNA) in the cells and tissues or after different forms of treatments (irradiation, chemotherapeutic drugs, etc.). These studies are often led by biologists or engineers, each approaching the question from their respective disciplines, and they have shown the feasibility of studying individual parameters. However, integrating these parameters to address broader questions such as point-of-care and personalized medicine or evaluating off-target effects of treatments in other organ systems has not yet been fully achieved. There is a need for long-term investment and collaboration among multidisciplinary teams in academia and industry to fulfill the innate potential of microfluidics for successful translation from preclinical findings to clinical decision-making in cancer diagnosis and treatment.

## Figures and Tables

**Table 1:** Examples of some commercially available microfluidic-based devices for cancer screening.

Company/Device	Analyte/Sample	Description	Ref.
ANGLE/Parsortix^®^ (Surrey, UK)	CTCs/Blood	- Single-use microfluidic cassette to capture and harvest CTCs - FDA cleared - Validated for metastatic breast cancer	[71]
AZAR innovations (Utrecht, The Netherlands.), Motamed Breast Cancer Research Centre, Eindhoven University of Technology	CTCs/Blood	- Micropillar array-based microfluidic chip (MPA-chip) - Validated for breast cancer	[72]
Menarini Silicon Biosystems (Bryn Athyn, PA, USA)/CELLSEARCH^®^ CTC test	CTC/Blood	- US-FDA 510(k) cleared in vitro diagnostic (IVD) test - Validated for epithelial cancers (breast, colorectal, prostate)	[73]

**Table 2:** Studies employing liquid biopsy samples for early detection and screening of head HNCs using microfluidic-based approaches.

	ctDNAs	CTCs	Exosomes
Location	Blood or Saliva	Blood	Blood, Saliva, Urine
Method of detection/screening /isolation	- Digital droplet PCR (ddPCR) [106,107] - Whole genome sequencing [108] - tagged-amplicon deep sequencing (TAm-Seq) [109]	Label dependent: - Positive/negative selection of antibodies (staining of EpCAM, CK, EGFR) [110,111] Label independent: - Size- or deformed-based separation [112,113] - Dielectrophoresis [114] - Density gradient separation [115]	Label dependent: - Staining of CD63 [116] Label independent: - Size exclusion chromatography [117] - Density gradient separation [118] - Immunoaffinity [119]
Sensitivity and specificity	- High/variable sensitivity - High/variable specificity	- Low sensitivity - Variable specificity	- High sensitivity - Low specificity
Other relevant characteristics	- Can detect tumor genetic mutations [120,121] - ctDNAs assays available for other types of cancers but none for HNC (lung, colorectal, pancreatic) [122,123]	- FDA-available approved clinical CTC assay for HNC (i.e., CellSearch) [124,125] - Difficult to detect and isolate from whole blood samples [126,127] - Difficult to capture tumor heterogeneity [128]	- Non-invasive and can be collected from various bodily fluids - Absence of clinical trials [129] - Difficult to capture tumor heterogeneity [129,130]
